# Early Versus Delayed Diuretic Administration and Urine-Guided Strategies in Acute Decompensated Heart Failure: A Systematic Review of Clinical Outcomes

**DOI:** 10.7759/cureus.89408

**Published:** 2025-08-05

**Authors:** Rabeet Muzammil, Ahmad Mohammad, Muhammad Hammad, Adeel Ahmed, Aadil Hussain, Wardah Rashid, Rabia Yousaf, Shivam Singla, Bhavna Singla, Osatohanmwen Ekomwereren, Francis Asante Baadu, Ahmad Irshad

**Affiliations:** 1 Cardiology, Faisalabad Medical University, Faisalabad, PAK; 2 Internal Medicine, Hurley Medical Center, Flint, USA; 3 Emergency Medicine, Aria Institute of Medical Sciences, Quetta, PAK; 4 Emergency Medicine, Dow University of Health Sciences, Karachi, PAK; 5 Internal Medicine, DHQ Teaching Hospital, Gujranwala, PAK; 6 Internal Medicine, Chandka Medical College, Larkana, PAK; 7 Internal Medicine, Khawaja Muhammad Safdar Medical College, Lahore, PAK; 8 Internal Medicine, Shifa International Hospitals Limited, Islamabad, PAK; 9 Internal Medicine, TidalHealth Peninsula Regional, Salisbury, USA; 10 Internal Medicine, Erie County Medical Center, Buffalo, USA; 11 Trauma and Orthopaedics, Royal Shrewsbury Hospital, Shrewsbury, GBR; 12 Internal Medicine, Komfo Anokye Teaching Hospital, Kumasi, GHA; 13 Internal Medicine, Combined Military Hospital, Muzaffarabad, PAK

**Keywords:** acute decompensated heart failure, clinical outcomes, early intervention, loop diuretics, natriuresis, systematic review, urine-guided therapy

## Abstract

This systematic review explores the impact of diuretic timing and strategy on outcomes in patients with acute decompensated heart failure (ADHF). A total of seven studies were included, comprising randomized controlled trials (RCTs), pre-specified sub-analyses, and observational data. Early administration of intravenous loop diuretics, particularly within the first 60 to 90 minutes of hospital arrival, was generally associated with improved short-term outcomes, including reduced in-hospital and 30-day mortality. Furthermore, guided diuretic strategies using urine sodium or urinary biomarkers showed promise in enhancing decongestion efficiency and predicting therapeutic response, although long-term benefits remain uncertain. Despite some heterogeneity in study design, timing definitions, and outcome measures, this review underscores the clinical significance of prompt and tailored diuretic therapy. These findings highlight the need for timely intervention and more personalized management strategies in ADHF, while also identifying gaps for future large-scale trials.

## Introduction and background

Acute decompensated heart failure (ADHF) represents a common and critical cause of hospital admission among patients with chronic heart failure, often presenting with signs of volume overload such as pulmonary congestion and peripheral edema [1]. Rapid and effective management of congestion is central to improving patient outcomes, reducing symptom burden, and shortening hospital stays. Intravenous loop diuretics remain the mainstay of initial therapy for volume relief in ADHF, yet the optimal timing of their administration has been a subject of ongoing investigation [2].

The pathophysiological cascade in ADHF involves a complex interplay between neurohormonal activation, renal dysfunction, and increased venous pressures. Delay in diuretic therapy may exacerbate these mechanisms, potentially leading to worsened hemodynamics, prolonged hospital stays, increased risk of organ dysfunction, and elevated mortality [3]. Conversely, early and aggressive decongestion may alleviate symptoms, restore hemodynamic stability, and improve end-organ perfusion. While guidelines recommend prompt diuretic administration, the precise window within which early therapy confers maximum benefit remains unclear [4].

Recent clinical studies and registries have attempted to characterize the relationship between time-to-diuretic administration and clinical outcomes. In addition, evolving strategies that utilize natriuresis-guided protocols or urine chemistry-based adjustments aim to tailor diuretic therapy early in the hospitalization course. However, variation in practice patterns and inconsistent definitions of "early" therapy have contributed to heterogeneity in results [5,6]. The objective of this study is to systematically evaluate and compare the outcomes of early versus delayed initiation of diuretic therapy in patients hospitalized with ADHF, with a focus on mortality, rehospitalization, renal function, and length of hospital stay.

## Review

Materials and methods

Search Strategy and Databases

This systematic review was conducted in accordance with the PRISMA (Preferred Reporting Items for Systematic Reviews and Meta-Analyses) guidelines [7] to ensure a transparent and reproducible methodology. A comprehensive literature search was performed across major biomedical databases, including PubMed, Scopus, Web of Science, and Cochrane Central Register of Controlled Trials (CENTRAL). The search focused on identifying relevant randomized controlled trials (RCTs) published within the last five years, up to June 2025, in the English language only. Boolean operators and MeSH terms were utilized to optimize sensitivity, with key search terms such as “acute decompensated heart failure”, “diuretic therapy”, “loop diuretics”, “timing”, “early”, “delayed”, “natriuresis-guided”, “urine sodium”, and “outcomes”

Eligibility Criteria

Studies were selected based on the PICO (Population, Intervention, Comparator, Outcomes) framework [8]. The population (P) included adult patients (≥18 years) hospitalized with ADHF. Interventions (I) involved early diuretic therapy or guided diuretic strategies (e.g., natriuresis- or urine chemistry-based titration). Comparators (C) were delayed diuretic administration or standard care without biomarker guidance. Primary outcomes (O) of interest included short-term mortality (in-hospital or 30-day), heart failure rehospitalization, worsening renal function, natriuresis efficacy, and symptom improvement. Only RCTs and high-quality pre-specified sub-analyses were included; observational or retrospective studies were only used to provide context in the discussion but were not formally synthesized.

Study Selection and Data Extraction

Two independent reviewers screened the titles and abstracts, followed by full-text reviews of potentially eligible studies. Discrepancies were resolved by discussion and, if necessary, consultation with a third reviewer. A structured data extraction form was used to retrieve study characteristics, including author, year, sample size, study design, diuretic strategy, time definitions for early or delayed administration, and reported outcomes. Only studies meeting all inclusion criteria were synthesized in the final review.

Risk of Bias and Quality Assessment

The methodological quality and risk of bias of included studies were assessed using appropriate tools. For RCTs, the Cochrane Risk of Bias tool (RoB 2) [9] was employed. For any included non-randomized trials or pre-specified observational analyses, the ROBINS-I tool [10] (Risk Of Bias In Non-randomized Studies - of Interventions) was used. Domains assessed included randomization, allocation concealment, blinding, attrition, selective reporting, and other biases. Only studies rated as low or moderate risk were included in the data synthesis.

Data Synthesis and Analysis

Given the heterogeneity in outcome definitions, intervention protocols, and diuretic strategies, a qualitative synthesis approach was adopted. Meta-analysis was not performed due to variability in clinical endpoints and methodological designs. Results were summarized narratively, with a focus on the timing of diuretic initiation, guided therapy protocols, and corresponding short-term and long-term outcomes. Studies were further stratified based on intervention type and presence of urine sodium-guided strategies.

Results

Study Selection Process

**Figure 1:**
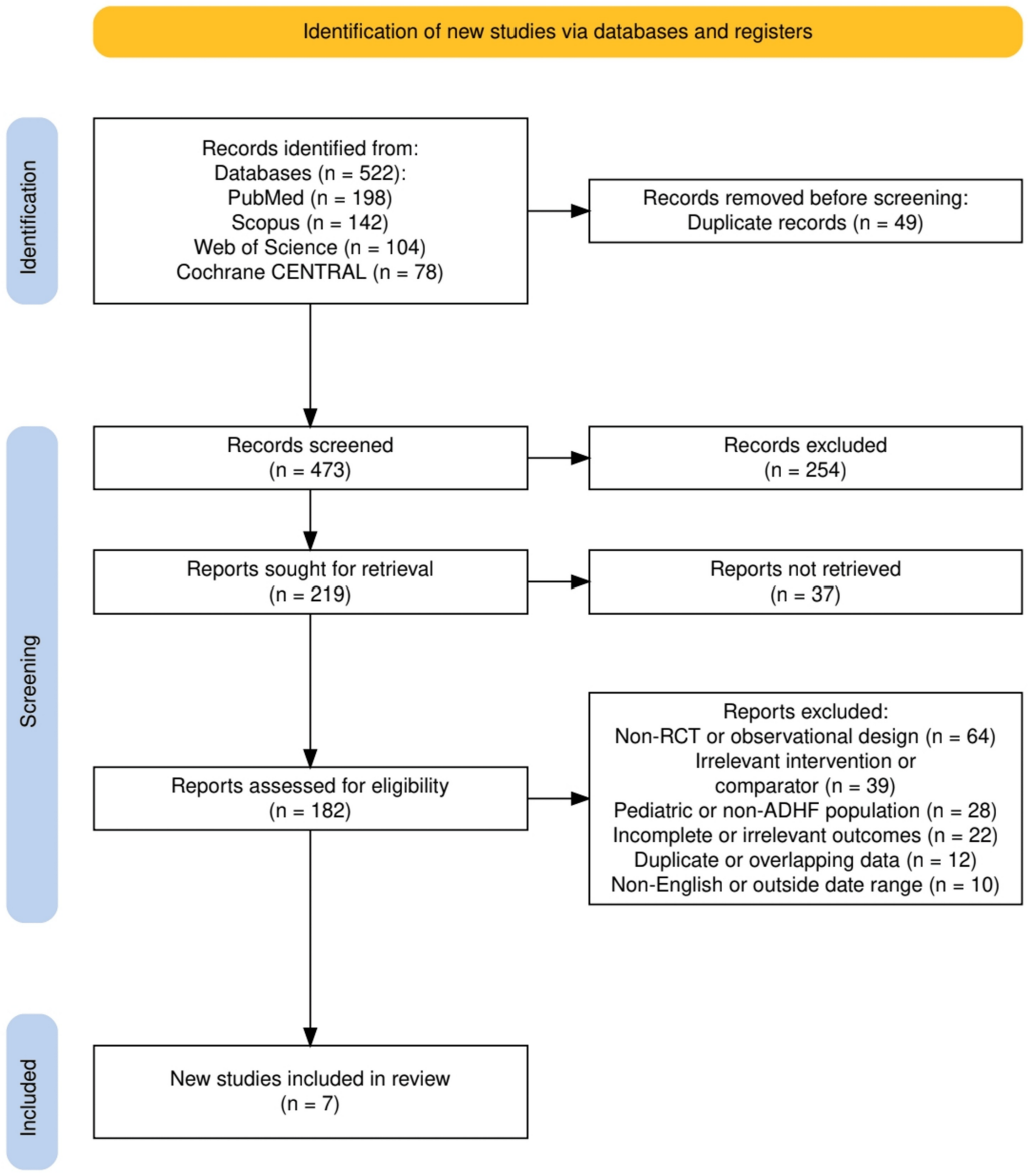
The PRISMA (Preferred Reporting Items for Systematic Reviews and Meta-Analyses) flow diagram represents the study selection process.

A total of 522 records were initially identified through database searches, as shown in Figure 1, including PubMed (n = 198), Scopus (n = 142), Web of Science (n = 104), and Cochrane CENTRAL (n = 78). After removing 49 duplicates, 473 records were screened. As shown in Figure 1, 219 reports were sought for retrieval, with 182 assessed for eligibility and ultimately seven studies meeting the inclusion criteria for this systematic review.

Characteristics of the Selected Studies

As summarized in Table 1, the included studies span a range of methodologies, from large observational registries like REPORT-HF (n = 15,078) [11] to smaller RCTs such as the DRAIN trial [12] (n = 80). These studies evaluated both the timing of diuretic administration and guided titration strategies, using metrics like spot urinary sodium levels and renal function. While most trials focused on short-term outcomes such as natriuresis, symptom relief, and 30-day mortality or rehospitalization, the findings consistently highlight the potential benefits of early or personalized diuretic strategies in managing ADHF.

**Table 1:** Summary of the included studies in the review. IV: intravenous, IQR: interquartile range, RCT: randomized controlled trial, HF: heart failure, SOC: standard of care, UNa⁺: urinary sodium, eGFR: estimated glomerular filtration rate, WRF: worsening renal function, Cr: creatinine, NT-proBNP: N-terminal pro–B-type natriuretic peptide, mEq/L: milliequivalents per liter, kg: kilogram, DRAIN: Diuretic Response Assessment in Acute Decompensated Heart Failure, PUSH-AHF: Personalized Urine Sodium-based Diuretic Strategy in Acute Heart Failure, DECONGEST: Diuretic Efficiency Comparison to Optimize Natriuresis in Guiding Early Strategy Trials, REPORT-HF: International Registry to Assess the Presentation and Treatment of Acute Heart Failure Patients, ASCEND-HF: Acute Study of Clinical Effectiveness of Nesiritide in Decompensated Heart Failure, ESCALATE: Evaluation of Sodium and Creatinine Algorithm for Loop Diuretic Titration and Effectiveness

Study (author, year)	Study design	Sample size	Diuretic strategy (early vs. delayed)	Time definition (early vs. delayed)	Primary outcomes measured	Key findings / protocol summary
Ouwerkerk et al., 2023 (REPORT-HF) [11]	Observational registry study	15,078	Time to IV furosemide administration	Median = 67-minute post-arrival (IQR 17–190 mins)	In-hospital mortality, 30-day mortality	Delay associated with increased 30-day mortality
Galluzzo et al., 2020 (DRAIN Trial) [12]	Randomized controlled trial	80	Stratified post-IV furosemide response (high UNa⁺ >50 vs. low UNa⁺ ≤50 mEq/L)	Spot urinary sodium measured two hours after IV furosemide	Diuretic response (urine output, weight change), NT-proBNP trends, incidence of WRF	Low natriuresis at two hours predicted poor response: lower urine output (2275 vs. 3849 mL), less weight loss (1.55 vs. 3.55 kg), higher WRF (32% vs. 10%), and NT-proBNP increased rather than decreased.
Fudim et al., 2021 (ASCEND-HF) [13]	Retrospective analysis of RCT	5,738	Early diuretic strategies (IV continuous vs. bolus vs. oral)	Stable regimen during the initial 24 hours	30-day mortality or HF rehospitalization; 180-day mortality	Route did not impact outcomes; supported early IV initiation
Ter Maaten et al., 2023 (PUSH-AHF) [14]	Pragmatic RCT	310	Natriuresis-guided diuretic titration vs. SOC	Spot UNa+ monitoring at fixed timepoints; escalation if <70 mmol/L	24-hour natriuresis; 180-day all-cause mortality or HF rehospitalization	Higher natriuresis in the guided arm; no difference in mortality or rehospitalization
Damman et al., 2024 (PUSH-AHF Sub) [15]	Pre-specified analysis from the RCT	309	Natriuresis-guided vs. SOC, stratified by renal function	eGFR measured at baseline, 72 hours, discharge	Interaction of renal function with 24-hour natriuresis and 180-day outcomes	Efficacy sustained even in low eGFR; WRF more common but transient and not harmful
Cox et al., 2023 (ESCALATE) [16]	Randomized controlled trial	450 (planned)	Urine chemistry-guided diuretic titration vs. usual care	Until IV diuresis completion; urine Na+ and Cr-based algorithm	Days of benefit (symptoms + IV diuresis/hospital-free days) over 14 days	First RCT testing full-course urine-chemistry-guided diuretic titration. Results awaited.
Vanhentenrijk et al., 2025 (DECONGEST) [17]	Pragmatic RCT protocol, multicenter	104 (planned)	Early combination diuretics guided by post-diuretic UNa+ monitoring	Serial post-diuretic UNa+ assessments until decongestion or UNa+ ≤ 80 mmol/L	Composite: 30-day survival, days alive/out of hospital, natriuretic peptide change	Testing intensive strategy with bolus loop diuretics, acetazolamide, and full nephron blockade; trial is protocol phase; useful for therapeutic context and future RCT justification

Risk-of-Bias Assessment

As shown in Table 2, the included studies exhibited varying levels of risk of bias depending on their design and methodology. RCTs generally showed low risk, particularly those with pre-specified protocols, proper randomization, and blinded outcome assessments. Observational and retrospective analyses carried a moderate risk due to potential confounding factors and selection bias inherent to non-randomized designs. Studies still in the protocol or ongoing phase were assessed with some concerns due to the absence of complete data and reliance on projected outcomes. Overall, the quality of evidence ranged from low to moderate, reinforcing the need for cautious interpretation.

**Table 2:** Risk-of-bias assessment of the included studies in the review. RCT: randomized controlled trial, RoB 2: Cochrane Risk of Bias Tool 2.0, ROBINS-I: Risk Of Bias In Non-randomized Studies of Interventions, AHF: acute heart failure, HF: heart failure, REPORT-HF: International Registry to Assess Medical Practice with Longitudinal Observation for Treatment of Heart Failure, ASCEND-HF: Acute Study of Clinical Effectiveness of Nesiritide and Decompensated Heart Failure, PUSH-AHF: Personalized Ultrafiltration Strategy in Acute Heart Failure, ESCALATE: Effectiveness of Early Ultrafiltration in Decompensated HF, DECONGEST: Decongestion-Guided Strategy in Acute Heart Failure, DRAIN: Diuretic Resistance Assessment and Intervention Network

Study (author, year)	Study design	Tool used	Overall risk of bias	Key risk considerations
Ouwerkerk et al., 2023 (REPORT-HF) [11]	Observational registry study	ROBINS-I	Moderate	Confounding due to patient condition severity; selection bias possible due to registry nature.
Galluzzo et al., 2020 (DRAIN Trial) [12]	Randomized controlled trial	RoB 2	Moderate	Randomization adequate, but possible detection and attrition bias; small sample size limits robustness.
Fudim et al., 2021 (ASCEND-HF) [13]	Retrospective analysis of RCT	ROBINS-I	Low to moderate	Risk from retrospective analysis; selection bias minimized due to initial RCT randomization.
Ter Maaten et al., 2023 (PUSH-AHF) [14]	Pragmatic RCT	RoB 2	Low	Well-randomized with good adherence to protocol; outcome assessment blinded.
Damman et al., 2024 (PUSH-AHF Sub) [15]	Pre-specified analysis from RCT	RoB 2	Low	Subgroup analysis pre-specified; consistent measurement approach; outcome reporting reliable.
Cox et al., 2023 (ESCALATE) [16]	Randomized controlled trial	RoB 2	Some concerns	Protocol published; trial ongoing; risk related to outcome measurement and incomplete data reporting.
Vanhentenrijk et al., 2025 (DECONGEST) [17]	Pragmatic RCT protocol	RoB 2	Some concerns	Still in protocol stage; risk due to lack of results and reliance on planned methods.

Discussion

Early Diuretic Administration and Time-Sensitive Outcomes

Our systematic review demonstrates that early and physiologically guided diuretic strategies in ADHF significantly influence short-term clinical outcomes. The largest observational dataset, REPORT-HF (Ouwerkerk et al., [11]), involving over 15,000 patients, revealed that delayed administration of intravenous loop diuretics was associated with a statistically significant increase in 30-day mortality. This finding reinforces the time-sensitive nature of initiating decongestive therapy and echoes long-standing clinical concerns regarding therapeutic inertia in ADHF management.

Further supporting this, the ASCEND-HF trial (Fudim et al., [13]), involving 5,738 patients, found that while the method of diuretic delivery (bolus vs. infusion) did not significantly affect mortality, early intravenous initiation contributed to better volume control and symptom relief. These results collectively underscore the importance of timing over mode of administration and suggest that early therapeutic intervention, even within the first few hours, may have a decisive impact on prognosis.

Guided Strategies: Urinary Sodium and Natriuresis as Precision Tools

Beyond timing alone, several trials explored the potential of individualized, physiology-guided diuretic therapy. The PUSH-AHF trial [14] (Ter Maaten et al., 2023) is particularly noteworthy in this regard. It showed that natriuresis-guided treatment significantly increased 24-hour sodium excretion, a key surrogate of decongestion, without increasing the risk of adverse outcomes such as mortality or readmission. This suggests not only safety but also potential efficacy of personalized decongestive strategies.

Similarly, the DRAIN trial (Galluzzo et al., [12]) emphasized the clinical value of early spot urinary sodium measurements. Patients with low urinary sodium (≤50 mEq/L at two hours post-furosemide) exhibited a markedly poorer diuretic response, evidenced by significantly lower urine output (2,275 mL vs. 3,849 mL, p < 0.001), reduced weight loss (1.55 kg vs. 3.55 kg, p < 0.001), a higher incidence of worsening renal function (32% vs. 10%, p = 0.02), and rising NT-proBNP levels. These data establish low urinary sodium as a reliable, early predictor of suboptimal diuretic efficacy, with important implications for clinical decision-making and escalation of therapy.

Advancing Beyond Traditional Paradigms

In contrast to earlier meta-analyses and registry data, which focused largely on the route or dose of diuretics, our review emphasizes an evolving paradigm centered on response-guided therapy [18]. PUSH-AHF [15] and DRAIN [12] extend this narrative by introducing urine-based biomarkers as actionable tools, offering an objective and quantifiable measure to tailor treatment. This evolution aligns with the broader shift in cardiology toward precision medicine, an approach that individualizes therapy based on physiologic and biochemical markers rather than relying solely on signs and symptoms.

Importantly, these findings challenge the traditional volume-centric models endorsed by current guidelines. While both European and American guidelines advocate early decongestion, they do not yet fully integrate urine chemistry as a guiding tool. Our synthesis suggests that biochemical metrics such as spot UNa⁺ or 24-hour natriuresis may offer an additional layer of control, particularly useful in managing complex or refractory patients.

Pathophysiological Justification for Early and Guided Decongestion

Early initiation of diuretics mitigates the cascade of hemodynamic and neurohormonal alterations triggered by persistent congestion. Delays in volume removal maintain elevated left ventricular filling pressures, exacerbate pulmonary and systemic congestion, and accelerate renal injury [19]. This ongoing strain activates the renin-angiotensin-aldosterone system (RAAS) and sympathetic nervous system, which collectively impair natriuresis and promote myocardial and renal remodeling [20].

Urine sodium-guided strategies allow for real-time assessment of renal tubular response to loop diuretics. Recognizing diuretic resistance early enables clinicians to intensify therapy before irreversible renal compromise occurs. By adopting a physiology-first framework, clinicians can potentially enhance the natriuretic effect, minimize nephrotoxicity, and optimize congestion relief [21].

Heterogeneity Across Study Designs and Populations

The included studies showed considerable heterogeneity in study design, patient selection, and outcome measures, which has both strengths and limitations. Trials such as PUSH-AHF [15] and DECONGEST [17] were prospective and pragmatic, while others like REPORT-HF [11] offered real-world registry data with broader patient capture. Timing definitions for “early” intervention ranged from ≤2 hours to ≤24 hours, and patient characteristics varied in age, baseline eGFR, sodium levels, and comorbidities.

These differences complicate direct comparisons or meta-analysis but also enrich the evidence base by providing a multi-faceted view of diuretic strategies across both controlled and routine care settings. Importantly, this breadth supports external validity and informs practical application across different clinical environments.

Strengths and Limitations of the Review

This review's strength lies in its dual focus on both timing and individualized strategies of diuretic therapy, an intersection rarely emphasized together in previous reviews. By including high-quality randomized trials alongside real-world registries, we aimed to balance internal and external validity. Methodological rigor was ensured through standardized bias assessment using ROB2 and ROBINS-I tools.

Nonetheless, some limitations warrant acknowledgment. Trials such as DRAIN [12] and DECONGEST [17] involved small sample sizes or short follow-up periods. Some protocols remain unpublished, limiting the availability of outcome data. In addition, this review excluded non-English language studies and did not conduct a pooled meta-analysis due to heterogeneity, potentially introducing selection and publication biases.

Clinical Implications for Practice

Our findings support the urgent initiation of intravenous loop diuretics in emergency or inpatient settings for ADHF, reinforcing existing guidelines. More importantly, the incorporation of urine sodium and natriuresis monitoring offers a novel, evidence-backed method for optimizing decongestive therapy. These strategies are especially relevant for patients at high risk of diuretic resistance, renal deterioration, or poor response to standard care [22].

From a systems perspective, emergency departments and intensive care units should consider incorporating spot UNa⁺ or urine output data into protocolized decision-making. This could help clinicians avoid underdosing or premature transition to second-line therapies and enable earlier identification of patients requiring ultrafiltration or adjunctive measures [23].

Future Directions and Research Gaps

This review highlights several research gaps deserving of further exploration. While trials like ESCALATE [16] and DECONGEST [17] are promising, the field lacks large, multicenter RCTs assessing long-term outcomes such as 6- or 12-month mortality, heart failure readmissions, and sustained renal function. Further research should also evaluate the cost-effectiveness and implementation logistics of guided therapy models in diverse health systems.

Innovative technologies, including artificial intelligence tools to predict diuretic response and remote urine chemistry monitoring, represent emerging frontiers. Future work must also address the training and workflow adjustments required to operationalize precision-guided strategies at the bedside and in resource-constrained settings.

## Conclusions

This review underscores the clinical importance of both the timing and strategy of diuretic administration in the management of ADHF. Synthesizing data from randomized trials and large observational cohorts, we demonstrate that early initiation of intravenous loop diuretics, especially when guided by real-time natriuretic markers such as urine sodium, is associated with improved decongestion efficiency and potentially better short-term outcomes. While heterogeneity exists in trial design and implementation, the emerging consistency in favor of guided, personalized therapy marks a significant evolution from conventional approaches. Our work is among the first to consolidate evidence on both timing and biomarker-driven strategies, offering a practical framework for optimizing acute heart failure management. It provides a strong rationale for clinicians to act swiftly and intelligently in initiating and titrating diuretic therapy and for researchers to design future trials that bridge current knowledge gaps.
